# Immunodominance of HIV-1 Specific CD8+ T-Cell Responses Is Related to Disease Progression Rate in Vertically Infected Adolescents

**DOI:** 10.1371/journal.pone.0021135

**Published:** 2011-07-19

**Authors:** Elizabeth R. Sharp, Christian B. Willberg, Peter J. Kuebler, Jacob Abadi, Glenn J. Fennelly, Joanna Dobroszycki, Andrew A. Wiznia, Michael G. Rosenberg, Douglas F. Nixon

**Affiliations:** 1 Division of Experimental Medicine, Department of Medicine, University of California San Francisco, San Francisco, California, United States of America; 2 Jacobi Medical Center, Bronx, New York, United States of America; Karolinska Institutet, Sweden

## Abstract

**Background:**

HIV-1 vertically infected children in the USA are living into adolescence and beyond with the widespread use of antiretroviral drugs. These patients exhibit striking differences in the rate of HIV-1 disease progression which could provide insights into mechanisms of control. We hypothesized that differences in the pattern of immunodomination including breadth, magnitude and polyfunctionality of HIV-1 specific CD8+ T cell response could partially explain differences in progression rate.

**Methodology/Principal Findings:**

In this study, we mapped, quantified, and assessed the functionality of these responses against individual HIV-1 Gag peptides in 58 HIV-1 vertically infected adolescents. Subjects were divided into two groups depending upon the rate of disease progression: adolescents with a sustained CD4%≥25 were categorized as having no immune suppression (NS), and those with CD4%≤15 categorized as having severe immune suppression (SS). We observed differences in the area of HIV-1-Gag to which the two groups made responses. In addition, subjects who expressed the HLA- B*57 or B*42 alleles were highly likely to restrict their immunodominant response through these alleles. There was a significantly higher frequency of naïve CD8+ T cells in the NS subjects (p = 0.0066) compared to the SS subjects. In contrast, there were no statistically significant differences in any other CD8+ T cell subsets. The differentiation profiles and multifunctionality of Gag-specific CD8+ T cells, regardless of immunodominance, also failed to demonstrate meaningful differences between the two groups.

**Conclusions/Significance:**

Together, these data suggest that, at least in vertically infected adolescents, the region of HIV-1-Gag targeted by CD8+ T cells and the magnitude of that response relative to other responses may have more importance on the rate of disease progression than their qualitative effector functions.

## Introduction

Host factors have a strong influence on the HIV-1-specific CD8+ T cell response and the consequent level of control exerted upon viral replication. Of particular importance are the genes contained within the MHC where diversity of class I driven responses have shown an advantage with regards to disease progression in HIV-1 disease. For example, individuals who are homozygous at any of the three HLA class I loci have a more rapid progression to AIDS, compared to those who are heterozygous at these alleles [1] suggesting an advantage to having a diverse repertoire of HIV-1-specific CD8+ T cell responses [2], [3], [4], [5], [6], [7].

HLA class I further influences immune responses by restricting the CD8+ T cell responses against a number of possible epitopes dictated by peptide binding specificities of the HLA allele. The size of each response generated by each epitope gives rise to a hierarchical order of responses [8], [9]. Within the hierarchy, the greatest response is defined as immunodominant, while the weaker responses are considered subdominant [10]. Development of immunodominant responses is dependent on many factors, including the kinetics of viral protein expression, the autologous sequence of the infecting virus in addition to the HLA alleles expressed by the individual [10]. The possession of certain immunodominant responses may be an important factor in establishing control over HIV-1 as has been observed in individuals expressing the “protective” alleles HLA-B*27 and -B*57 [8], [11], [12], [13].

Viral control may also be linked to CD8+ T cell responses against specific epitopes that afford a greater (or lesser) degree of protection for the host. This concept is strengthened by the consistent association of some HLA Class I alleles with HIV-1 disease progression rates; the association of HLA-B*27 and HLA-B*57 with long-term non-progression and HLA-B*35 with rapid disease progression [14], [15], [16], [17]. However, expression of an allele and subsequent response to a protective epitope alone is not alone sufficient to confer a disease progression pattern. In a study comparing the HIV-1-specific immune response between HLA-B*57 long term non-progressors (LTNPs) and HLA-B*57 typical progressors, the LTNPs focused more of their responses to peptides known to be HLA-B*57-restricted [18] suggesting the dominance of the dominance of immune response is also important.

The targeted viral gene product is an additional variable that may impact disease course. The presence of an HIV-1-Gag-specific response has been shown to be associated with a better clinical outcome. Several studies in chronically infected adult patients have shown that individuals whose immune response is preferentially targeted against Gag progress more slowly and/or have a lower viral load [3], [19], [20], [21]. Studies have shown that individuals who control their virus preferentially target Gag derived epitopes during acute infection, and this is also seen in individuals who express the protective alleles HLA-B*27 or HLA-B*57 [12], [22], [23]. Importantly, one of the few studies performed in perinatally infected infants showed that children with Gag-specific CD8+ T cell responses exhibited significantly lower viral loads than those that did not respond to Gag [24]. These data strongly suggest that T cell responses directed against the Gag protein are beneficial and may contribute to slower HIV-1 disease progression in both adults and children.

This study was performed to investigate the importance of immunodominant CD8+ responses in the clinical progression of perinatally HIV-1-infected adolescents using responses to HIV-1 Gag to interrogate the key parameters. We hypothesized that differences in the breadth, magnitude, polyfunctionality and immuondomination patterns of the HIV-1 specific CD8+ T cell response could explain differences in rate of progression.

## Results

### Subject Cohort Characteristics

**Figure 1:**
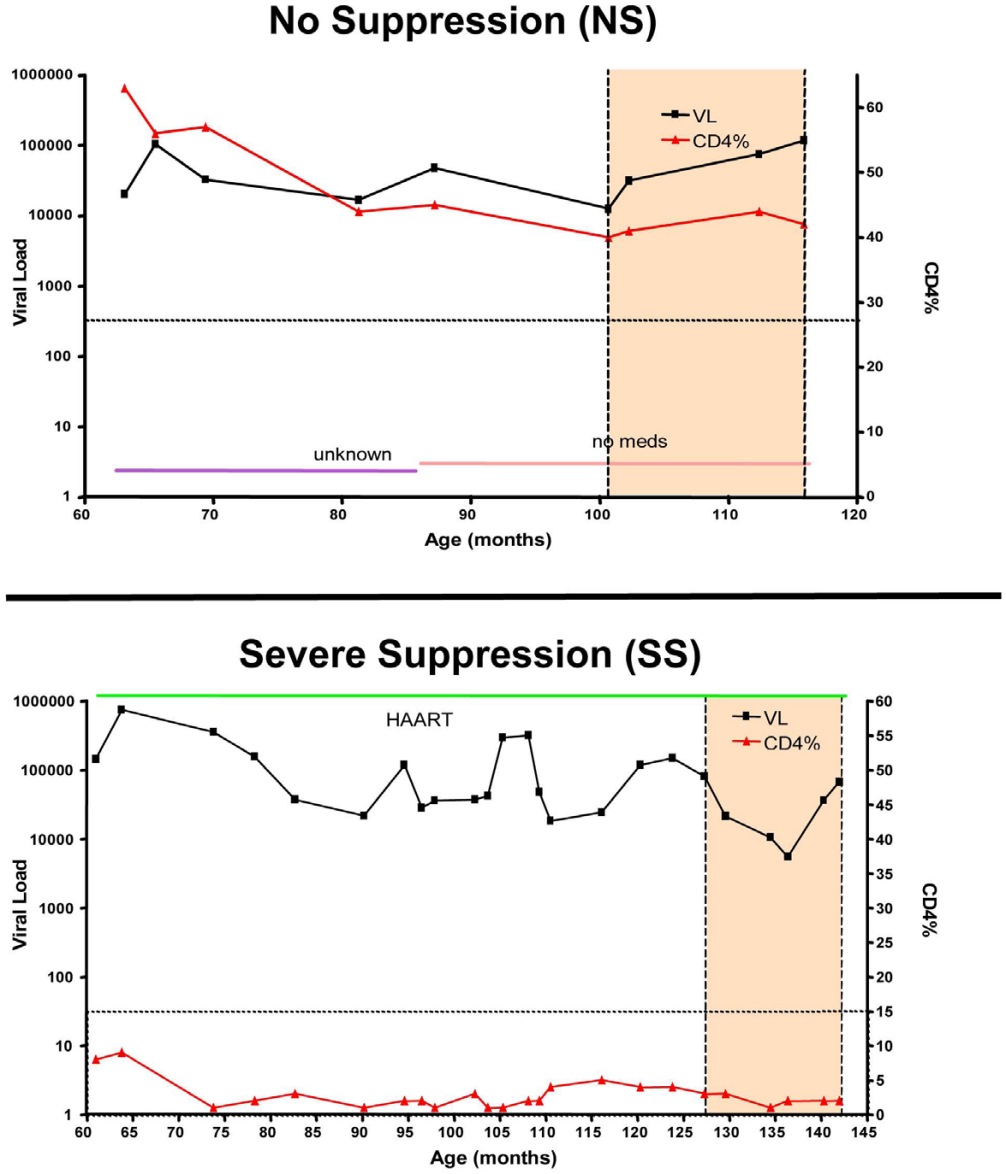
Categorization of subjects into two distinct disease progression groups. Two examples of representative patients categorized into either long-term survivors with No Immune Suppression (NS) (A) or Severe Immune Suppression (SS) (B). The dotted line is the boundary of the CD4% value of that progression group. The shading shows the window from within which PBMC samples were chosen for study.

Peripheral blood samples from 58 children and adolescents with vertically acquired HIV-1 were analyzed. Subjects were divided into two groups of immunologic progression based on CDC guidelines (NS and SS). The characteristics of both groups as well as the total cohort are displayed in table 1. The groups were overall well distributed with regards to age, sex and race although the SS group had a slightly higher percentage of females and the NS group a slightly higher percentage of African Americans. The mean ages for the NS and SS groups were 13.8 and 15.6 years respectively. An example of how timepoints were selected is in figure 1.

**Table 1:** Patient cohort characteristics.

Immunological Category	N	CD4%	LVL^a^	Age (y)	Sex	Race^b^
No immune suppression (**NS**; CD4%≥25)	30	29.8% (27.1; 34.8)	3.65 (2.85; 4.14)	13.8 (10.9; 16.6)	M = 15 F = 15	H = 9 AA = 20
Severe immune suppression (**SS**; CD4%<15)	28	8.25% (5.5; 11.5)	4.79 (4.37; 5.11)	15.6 (12.5; 18.1)	M = 11 F = 17	H = 13 AA = 13
**TOTAL**	58	24.5% (8.25; 31.0)	4.28 (3.61; 4.81)	14.3 (11.6; 17.52)	M = 26 F = 32	H = 22 AA = 33

a Log viral load.

b H = Hispanic; AA = African American; M = Male; F = Female; Race was not available for 1 subject in the NS group and 2 subjects in the SS group.

The numbers in parentheses represent 95% confidence intervals.

Viral loads between the two groups were different consistent with the difference in immune suppression status. None of the patients exhibited consistently undetectable viral loads indicating that all patients experienced some degree of viremia and consequently HIV–1 antigenic exposure. The NS group included more African-Americans than the SS group, but this was not statistically significant. Fifty six of the 58 subjects were on or previously on an antiretroviral treatment regimen with variable adherence levels; no gross differences in antiretroviral drug treatment regimen or adherence levels between the two clinical groups were observed.

### HLA Allele Distribution among Progression Groups and Associations with Clinical Characteristics

**Figure 2:**
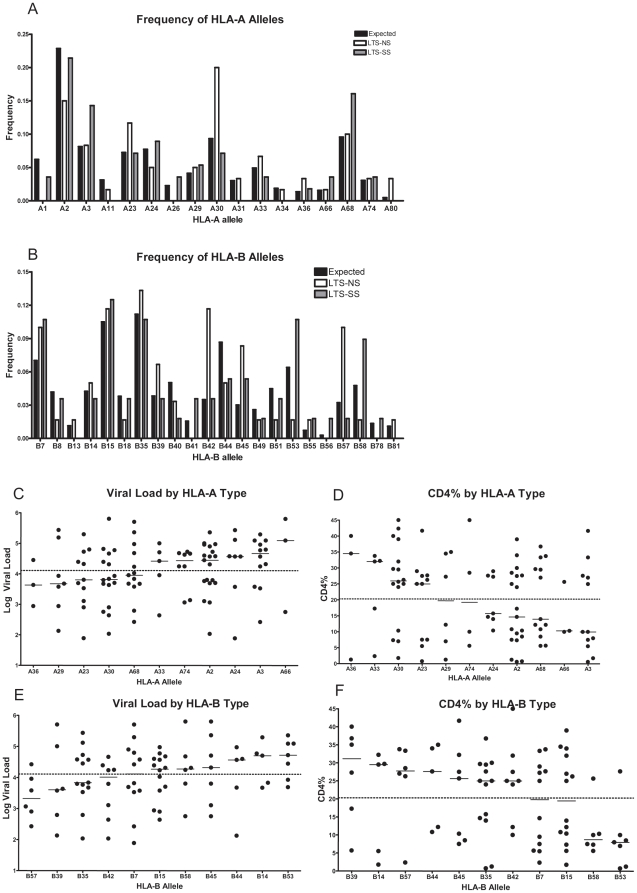
Distribution of HLA class I alleles in study Cohort. The frequency of expression of HLA class I A alleles (A) and B alleles (B) among the two progression groups in the cohort was compared to the expected frequency in a Hispanic and African American cohort (see methods). Associations between HLA Class I alleles and clinical characteristics are shown in panels C–F. Associations between log viral load (LCL) of all subjects and class I HLA-A alleles (C) and B alleles (D), are ordered from lowest LVL to highest. Associations between CD4% values and HLA Alleles (E) and B alleles (F) are ordered from highest CD4% to lowest. The solid line in each column is the median value for that HLA allele. The dotted line is the median value of the total cohort.

All subjects were HLA typed and revealed a wide range of HLA-A and HLA-B alleles among the two progression groups. The allele frequency of the cohorts were consistent with published data on a comparable population from the National Marrow Donor Program [25], figure 2.

Among the alleles expressed in this cohort, distribution differed amongst the NS and SS groups. The frequency of HLA-A*30, B*42, and B*57 was greater in the NS group whereas HLA-B*53 and B*58 were more common in the SS group. These associations did not reach statistical significance, but the observed trends suggest influences of HLA alleles on disease progression rate.

HIV-1 viral load and CD4% were inversely correlated as expected. Alleles which appeared with greater frequency in the NS cohort had lower overall viral loads and higher CD4% values (figure 2C–F). Certain HLA-B alleles such as B*57, B*35, B*39, and B*42 were associated with lower viral loads and higher CD4% levels as measured by the median overall viral load and CD4% respectively.

### Gag-specific CD8+ T cell Responses

The regions of Gag targeted by each group were examined by ELISPOT in 2 stages, looking first at overall reactivity to p17, p24 and p15 followed by reactivity to peptides within those regions where activity was observed. Five subjects (8.7% of the cohort), did not display any Gag-specific response in the first screening ELISPOT; 2 subjects displayed a Gag response, but there were insufficient samples for further experiments, leaving 51 subjects in the study.

**Figure 3:**
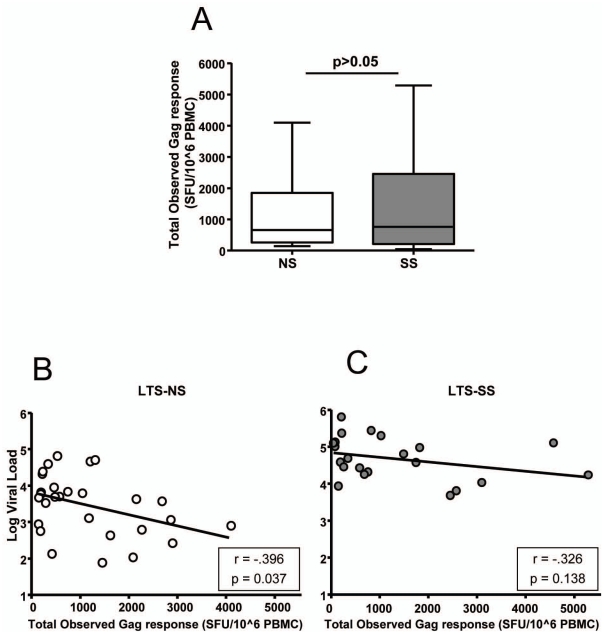
Association between Gag specific responses and disease progression. The magnitude of the total observed Gag specific responses (the sum of all the epitope- specific responses) is compared between the two groups in panel A. Correlations between LVL and the magnitude of the total observed Gag response are displayed for both progression groups in panels B and C.

Overall HIV-1-Gag-specific CD8+ T cell responses were summarized in order to test for gross differences between the groups. When pooling the total number of Gag responses, we did not observe a meaningful quantitative difference (figure 3A). We then looked for differences in the impact of the Gag-specific responses on viral load. There was a statistically significant, but modest negative correlation, between the overall magnitude of the Gag-specific CD8+ T cell responses and log viral load (LVL) (Spearman r = −0.396, p = 0.0369) in the NS group (figure 3B) but not in the SS group (figure 3C).

**Figure 4:**
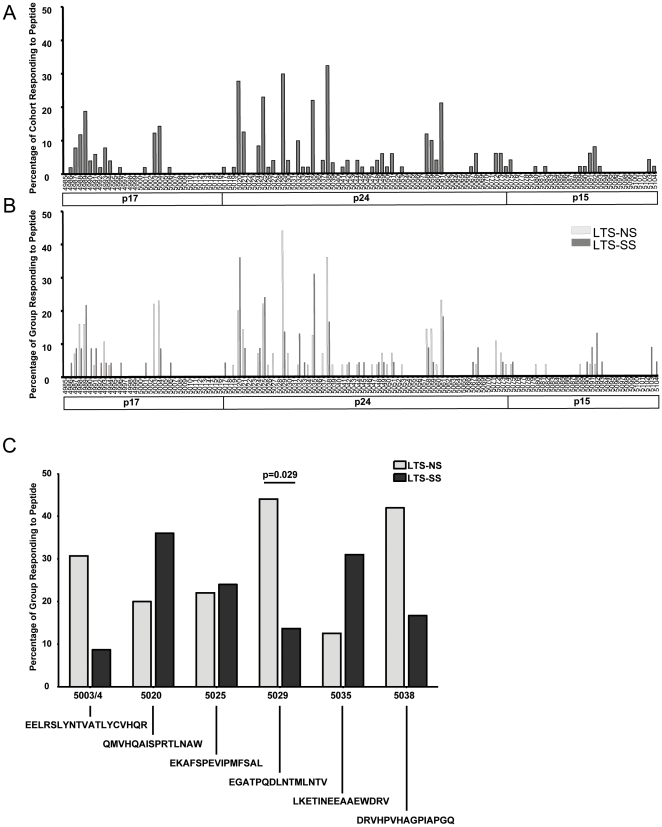
Areas of Gag targeted by CD8+ T cell responses. The frequency of recognition of individual single peptides are shown for the total cohort in panel A. The NIH AIDS Reagent peptides for HIV-1 Gag are represented by their peptide number. A comparison of the frequency of recognition of single peptides between the two progression groups is shown in panel B. The percentage of each group responding to 6 of the individual Gag peptides is shown in panel C.

Of the three subunits of Gag, the p24 region was the most frequently targeted area, with 100% of the NS subjects and 80% of the SS subjects recognizing this region. This was followed by p17, recognized by 66% of NS and 67% of SS subjects. The least recognized Gag subunit was p15, with only 24% of NS and 38% of SS subjects displaying a response to this region (data not shown). Overall, several areas were more frequently targeted than others (figure 4A).

### Specific Regions of Gag Targeted Unequally Between Groups

Once the larger regions of response were identified for each subject, the single peptides within the pool that an individual could respond to were predicted from data in the Los Alamos National Database CTL epitope maps. A second ELISPOT assay was performed using the single peptides (table 2), to identify a more precise estimate of the epitope targeted by the individual. Peptides 4988/4989, 5003/5004, 5020, 5025, 5029, 5038, 5061, and 5035 were the most frequently targeted. The peptide number, location, amino acid sequences are shown in table 2.

**Table 2:** Frequently Targeted Peptides.

Peptide Number	Location protein (aa–aa)	Amino Acid Sequence
4988/9	p17 (13–31)	LDRWEKIRLRPGGKKKYKL
5003/4	p17 (73–91)	EELRSLYNTVATLYCVHQR
5020	p24 (9–23)	QMVHQAISPRTLNAW
5025	p24 (29–43)	EKAFSPEVIPMFSAL
5029	p24 (45–59)	EGATPQDLNTMLNTV
5035	p24 (69–83)	LKETINEEAAEWDRV
5038	p24 (81–95)	DRVHPVHAGPIAPGQ
5061	p24 (173–187)	RAEQASQEVKNWMTE

Several peptides were targeted more frequently by one group than the other (figure 4B and C). Notably, peptide 5029 was recognized by significantly more NS subjects with an HLA allele able to present the peptide (44%), than SS subjects (13.6%) (p = 0.029, Fisher's exact test). Peptides 5003/4 and 5038 were also more frequently targeted by the NS group than SS (30.8% vs. 8.7% and 42% vs. 16.7% respectively) although these differences did not quite reach statistical significance (p = 0.07 for both). In contrast, peptides 5020 and 5035 were more frequently targeted by subjects in the SS group compared to the NS group (36% vs. 20% and 31% vs. 12.5%, respectively).

### B*57 and B*42 Restricted Epitopes are Highly Immunodominant

**Figure 5:**
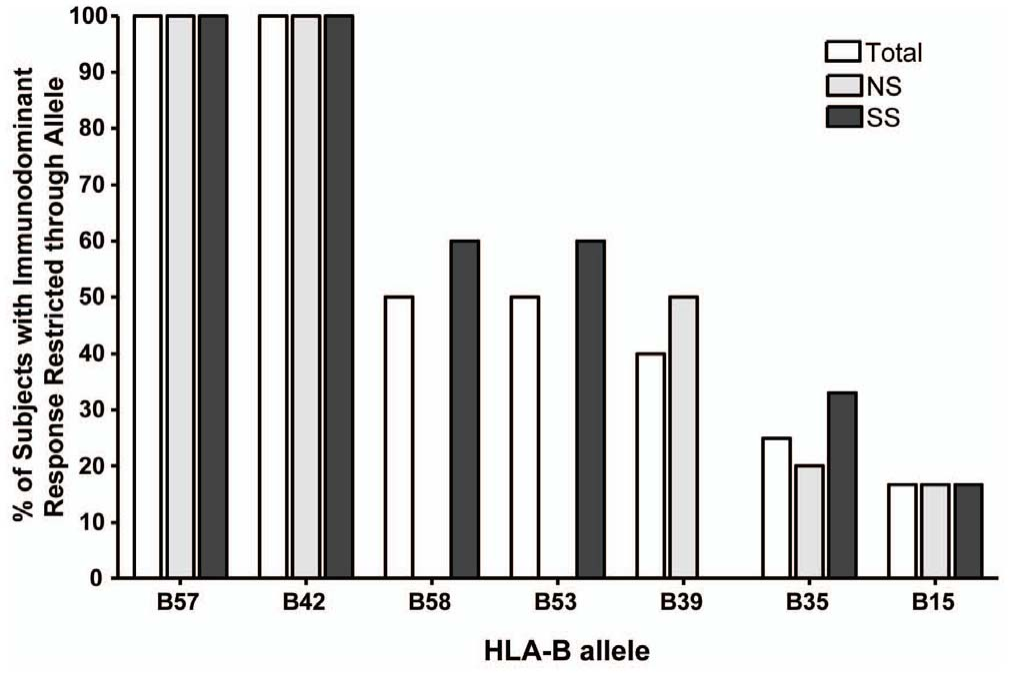
Immunodominance of CD8+ T cell responses restricted by HLA-B alleles. The frequency of several HLA-B alleles restricting the immunodominant response of individuals expressing that allele, shown for each group.

With the knowledge of each individual's HLA alleles and the regions of Gag they responded to, we could determine the HLA-restriction of the vast majority of responses using data from the Los Alamos National Database. Subjects who expressed the HLA- B*57 or B*42 alleles were more likely to restrict their highest magnitude (immunodominant) responses through peptides with known B*57 and B*42 epitopes suggesting a strong contribution of these alleles to immunodominance. In addition, such responses made up the great majority of the total Gag-specific response in these individuals (figure 5).

Eight subjects in this cohort expressed at least one HLA-B*42 allele and all eight restricted their immunodominant response through this allele (figure 5). B*42 restricted responses accounted for an average of 69% of the total observed Gag-specific response in these individuals. Similarly, five subjects expressed at least one HLA- B*57 allele and again, all 5 of these subjects had an immunodominant response restricted through B*57. In these subjects, B*57 restricted responses accounted for, on average, 84% of their total observed Gag-specific response.

### Comparison of Differentiation Profiles of Bulk and HIV-1-specific CD8+ T cells

**Figure 6:**
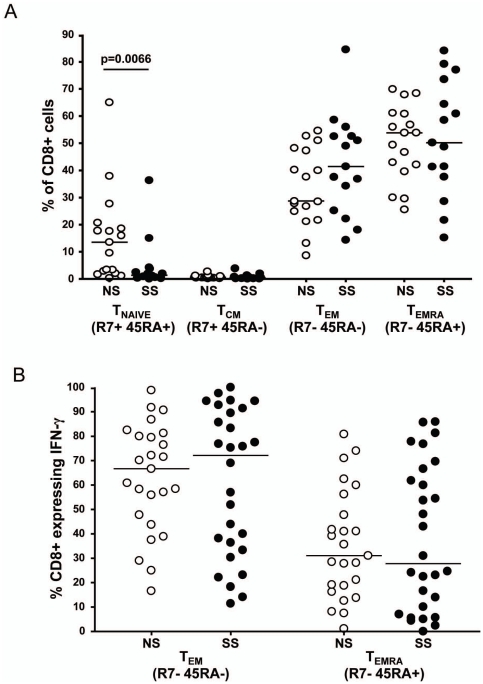
Comparison of differentiation profiles of Gag specific effector CD8+ T cells between progression groups. All Gag specific responses are shown in panel A. Only immuodominant responses (the response with the highest magnitude for each individual) are shown in panel B. The line in each column represents the median value. The differentiation phenotype is referenced beneath each column.

Surface and intracellular cytokine staining on 17 NS subjects and 15 SS subjects was performed following PBMC stimulation with the previously identified single Gag peptides. We hypothesized that a more fully differentiated T_EMRA_ cells in the NS subjects compared to SS subjects, both in the total CD8+ T cell population and in Gag-specific CD8+ T cells would be observed based on previous studies of HIV-1 infected adults [2], [26]. Surface staining of the total CD8+ T cell population revealed a significantly higher frequency of naïve T cells (CCR7+CD45RA+) in NS subjects (p = 0.0066). We observed a trend towards higher levels of T_EM_ (CCR7−CD45RA−) cells in the SS group although this was not significant (p = 0.2). There was no observable difference in the levels of T_CM_ (CCR7+CD45RA−) or T_EMRA_ (CCR7−CD45RA+) cells between the two groups (figure 6).

Intracellular cytokine staining allowed epitope-specific CD8+ T cells to be characterized and for the maturation profiles between the two groups to be compared. Initially, all epitope-specific responses were analyzed, regardless of immunodominance. No differences in the maturational profiles of epitope-specific CD8+ T cells between the two groups were observed (figure 6B). Subsequently, only the immunodominant response from each person was analyzed. Again, there were no statistically significant differences in the maturational profiles, although there seemed to be a trend towards higher levels of Gag-specific T_EMRA_ cells and lower levels of T_EM_ cells in the NS groups (p = 0.2 for both) (data not shown).

### Levels of Cytokine and Degranulation Molecule Secretion

**Figure 7:**
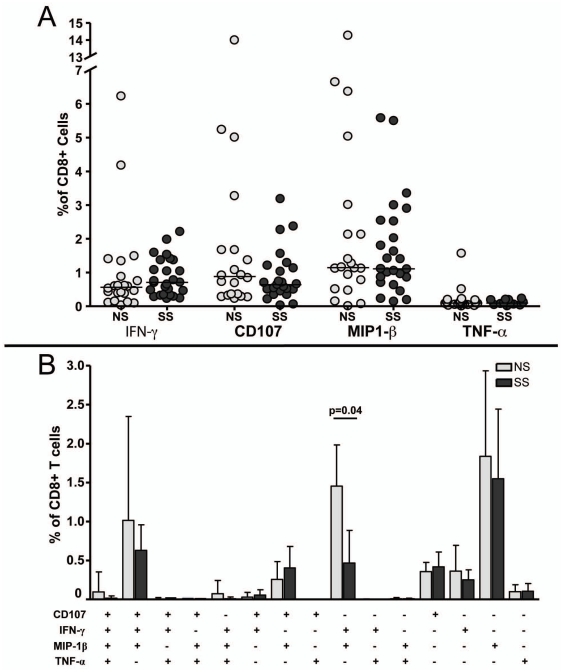
Comparison of cytokine secretion of HIV-1 Gag specific CD8+ T cells between progression groups. Secretion of single cytokines is shown in panel A. Functional profile of HIV-1 Gag specific CD8+ T cells is shown in panel B. (+) denotes a positive response for that particular cytokine. Gray bars represent NS subjects and black bars represent SS subjects. The solid bars represent the mean and the error bars represent the standard error of the mean.

Secretion of the cytokines IFN-γ, TNF-α, MIP-1β, and the degranulation marker CD107a/b were analyzed in order to assess the CD8+ T cell function. The secretion levels of these molecules were analyzed both singly, and in different multifunctional combinations. The frequencies of cells secreting any levels of the molecules did not differ significantly between the two progression groups (figure 7A). Overall, the frequency of cells secreting TNF-α was quite low, never reaching above 0.25% of total CD8+ T cells. The frequencies of cells secreting other cytokines varied widely, with MIP-1β being secreted by the most cells; a median of 1% total CD8+ T cells were found to secrete MIP-1β.

The vast majority of epitope-specific CD8+ T cells secreted only 1 or 2 cytokine(s) or degranulation marker, out of a possible 4 (figure 7B). A subset of Gag-specific CD8+ T cells did secrete IFN-γ, MIP-1β, and CD107a/b simultaneously (median of 0.6% of total CD8+), although there was no difference in the frequency of these cells between the two progression groups. There was a significant difference between the groups in the frequency of cells secreting IFN-γ and MIP-1β simultaneously, with NS subjects having a higher frequency of these cells (p = 0.04) than SS subjects. There appeared to be trend toward SS subjects possessing a higher frequency of cells secreting CD107+ and MIP-1β simultaneously than NS subjects, but this was not statistically significant (p = 0.13).

## Discussion

Several studies have suggested that qualitative characteristics of the HIV-1-specific CD8+ T cell response are associated with viral control and disease progression [2], [26], [27], [28], [29], [30]. Other studies have suggested that immunodominance patterns of HIV-1-specific CD8+ T cell responses are of great importance in establishing control of the virus and HIV-1 disease [11], [12], [20], [23], [31], [32], [33], [34], [35], [36], [37], [38], [39], [40]. We observed no difference in the magnitude or breadth of the Gag-specific CD8+ T cell response between the two groups, as measured by IFN-γ production, consistent with other studies [2], [3], [4], [5]. In contrast, there were several differences in the epitope regions of Gag that were targeted by CD8+ T cell responses. In bulk CD8+ T cell populations we observed a significantly higher frequency of naïve CD8+ T cells in the NS subjects (p = 0.0066) compared to the SS subjects, but no differences in any other CD8+ T cell subsets. The differentiation profiles and multifunctional capacity of Gag-specific CD8+ T cells, regardless of immunodominance, were similar between the progression groups. Together, these data suggest that, at least in perinatally infected adolescents, the region of Gag targeted by CD8+ T cells may have more importance to the rate of disease progression than qualitative features such as differentiation and multifunctionality.

There have been conflicting reports on the association between the breadth of the Gag-specific response and disease progression. Early studies showed a negative association between the breadth of the HIV-1-specific response and disease progression [19], [41], [42], while other studies have observed no relationship [2], [3], [4], [5]. Other investigations have found that individuals with putative protective HLA alleles have CD8+ T cell responses that predominantly target specific regions within the Gag protein during acute infection [12], [22], [23]. These data suggest that the overall breadth of response is not as important as which epitopes are targeted, specifically those regions in Gag that are restricted by protective alleles.

The peptide (5029) which was significantly targeted more frequently by NS relative to SS subjects (44% vs. 13.6%), contains the TL9 (TPQDLNTML) epitope, which has previously been identified as a peptide frequently targeted by HLA-B*42 restricted CD8+ T cell responses [43]. The TL9 epitope shares significant homology to the Mamu-A*01-restricted epitope CM9 (CTPYDINQM), which has been implicated in viral control in SIV-infected macaques [44], [45], [46]. This suggests that recognition of this area of Gag could be important in establishing immune responses that might be contributing to the slow disease progression in perinatally infected adolescents.

Of the various MHC Class I alleles in this study cohort, only HLA-B*57 and B*42 overwhelmingly restricted immunodominant responses in that 100% of subjects with at least one copy of these alleles restricted their immunodominant response through that allele. Interestingly, these two alleles occurred much more frequently within the NS group, suggesting that the immune response directed through these alleles plays a role in mediating slower disease progression. This finding is consistent with the effect of the HLA-B*57 allele on slow disease progression in adults [12], [18], but has not been demonstrated in a perinatally infected population. HLA-B*42-restricted responses have been noted for their immunodominance in a population with African ancestry [43], but have not previously been associated with slower disease progression.

Although this study suggests that immunodominance patterns are a critical component in the disease progression of perinatally HIV-1-infected adolescents, several caveats remain. The small number of subjects precludes us from applying these findings to the general HIV-1 infected pediatric population. These patients were not all receiving the same treatment regimen, but this is an inherent concern in studies where the subjects are drawn from heterogeneous clinic populations. We attempted to minimize this concern, by choosing subjects for the study who: 1) had not received HAART in the first two years of life; 2) had some levels of ongoing viral replication; and 3) were ARV-experienced (except for two patients). Both groups had generally similar treatment adherence rates to antiretroviral drugs, and variable adherence to ART is common in HIV-1 infected adolescents. We used the HXB-2 peptide set from the NIH as antigens, and this may both underestimate and potentially miss autologous epitopic responses. As we were limited with cell numbers, we focused on the HIV-1 Gag antigen, and responses to other HIV-1 proteins are important to consider [32].

Our study was well balanced overall for age with a range from 10 to 18 years between the 2 groups studied. Age of the subject could be contributing to the pattern of immunodominance. However, the age range is relatively narrow with most subjects developmentally considered adolescents. Therefore, age was considered to be less unlikely to yield meaningful relationships to immunodominance patterns and therefore no formal testing for the effect of age was conducted.

This study is one of the first to study the patterns of immunodominance and associations with disease progression in a cohort of perinatally infected adolescents. Moreover, this work focused on African American and Hispanic children, two populations that are greatly underrepresented in studies on the HIV-1-specific immune response. We find that in adolescents, as in adults [12], the immunodominance patterns appear to influence rates of disease progression. This influence seems to be mainly focused on the exquisite targeting of certain Gag epitopes, and not on the differentiation or cytokine secretion profiles of antigen-specific CD8+ T cells. These findings may be of importance to the field of pediatric HIV-1 immunology as well as the larger field of HIV-1 vaccine design.

## Materials and Methods

### Ethics Statement

The research involving human participants reported in this study was approved by the UCSF and AECOM institutional review boards, with the approval number H11613–19149. Informed consent was obtained for all subjects. All clinical investigation were conducted according to the principles expressed in the Declaration of Helsinki.

### Patient Sample Characteristics

Peripheral blood mononuclear cells (PBMC) from 58 subjects attending the Pediatric HIV clinic at Jacobi Medical Center in the Bronx, NY were selected based on availability of pre-existing cryopreserved samples and clinical characteristics that allowed them to be classified according to previously published CDC guidelines [47]. All subjects contracted HIV-1 by vertical transmission.

As a result of very limited cells in each sample (about 5–10 million cells per sample), and the need to perform three separate assays on each patient for this study, we pooled PBMC samples at three timepoints for each patient. We chose three consecutive samples for each patient, coming from three consecutive clinic visits. There was an average of 3.67 months between clinic visits. An example of how timepoints were selected can be found in figure 1.

### Subject Categorization

Because the CD4% value is less variable than the CD4+ T cell count in children, it is considered a more consistent marker of immunologic disease progression, and is used by the CDC to classify levels of progression [47]. Patients were categorized into two groups based on CD4% values, using guidelines set forward and published by the CDC [47]. Subjects with a sustained CD4%≥25 are considered long term survivors (LTS) with No Evidence of Immune Suppression (NS). Those with a sustained CD4%≤15 are considered as having Severe Immune Suppression (SS). We acknowledge that some of these subjects could be classified as having “mild” or “moderate” suppression, but we have given an overall classification of “severe” to help distinguish the group from those with a higher sustained CD4%. None of the subjects received HAART during the first two years of life. All of the patients, with the exception of 2 children, were either taking antiretrovirals (ARVs) or were ARV-experienced, but the vast majority had variable adherence levels. There were no gross differences in treatment regimen which included both protease inhibitors and RT inhibitors, or adherence levels between the two clinical groups.

### HLA typing

DNA was extracted from PBMC using a QIAamp DNA Mini kit (QIAGEN Inc., Valencia, Calif.). HLA typing used an amplification refractory mutation system with sequence-specific primers as described by the manufacturer (Invitrogen, Carlsbad, CA).

### Determining Epitope-specific Responses

Due to the limited cell numbers in samples, we determined the repertoire of epitope-specific CD8+ T cell responses using a two-stage ELISpot strategy. Antigenic stimulation was achieved through the use of 122 peptides, comprising the entire HIV-1 HXB2 Gag protein: 15 to 19-mers overlapping by 10 amino acids. Lyophilized peptides were obtained from the NIH AIDS Research and Reference Reagent Program and resuspended in DMSO and/or PBS. Initially, the individual peptides were combined to form 12 sequential pools of 10–13 peptides. PBMC reactivity was first screened with the 12 sequential pools at 2 µg/ml in an IFN-γ ELISpot in order to identify the broad regions of Gag that were being targeted by each patient.

After the determination of which pools each subject responded to, a targeted approach to identifying the individual peptides and epitopes each individual responded to, was pursued. Potentially reactive epitopes within a positive pool response were identified using the patients HLA type and documented HLA-specific epitope responses provided by the HIV-1 Molecular Immunology Database (Los Alamos National Database). A database that allows the rapid identification of epitope-containing peptides was also used, which can be applied in a HLA specific manner [48]. We then verified these predicted epitope-specific responses by performing a further IFN-γ ELISPOT, using single peptides, at 10 µg/ml, containing the predicted epitopes as antigenic stimulation. Comparisons of results were only made with data generated from similar peptide concentrations to minimize this as a confounding variable. Due to a limited number of cells, optimal epitope identification was not possible.

### Detection of Gag-specific CD8+ T-cells by IFN-γ ELISPOT

Quantification of HIV-1-specific T-cell responses using thawed viable PBMC was performed using the IFN-γ ELISPOT assay [49]. Briefly, each well of a 96-well plate (Millipore MAHAS4510, Bedfort, MA) was coated with 50 µl of anti-IFN-γ mAb (Mabtech, Stockholm, Sweden) at 5 µg/ml. After incubation, each well was washed and blocked with 10% FCS in RPMI (Cellgro). PBMC (1×10^5^–2×10^5^) were added to duplicate wells and peptides comprising the HXB2 sequence of Gag (AIDS Research and Reference Reagent Program, NIH) were added at 2 or 10 µg/ml to the cells. As a positive control, the mitogen phytohemagglutinin (PHA) was used at 4 µg/ml, and wells with only media added were used as a negative control. After overnight incubation (14–16 hours) at 37°C, plates were washed with phosphate-buffered saline (PBS). Biotinylated anti-IFN-γ mAb 7-B6-1 (Mabtech) was added at 1 µg/ml, and incubated at 37°C for 1 hr Plates were washed with PBS+0.1% Tween 20 and treated with streptavidin-bound alkaline phosphatase. After 1 h incubation, plates were washed with PBS+0.1% Tween 20 and developed using Alkaline Phosphatase Substrate Kit III (Vector Laboratories, Burlingame, CA). IFN-γ spot-forming units (SFUs) were visualized and counted using an AID EliSpot reader (Autoimmun Diagnostika GMBH, Germany). Spots were standardized to SFU/10^6^ PBMC. Spots formed in the presence of media alone were considered non-specific background and subtracted from the SFU in stimulated wells. A reading of 30 SFU/10^6^ PBMCs after the subtraction of non-specific background spots was considered positive, as previously defined by pediatric studies, using the same or a similar assay, performed by our lab [50], [51], [52], [53]. We defined the immunodominant response as the strongest response detected in the ELISpot assay.

### Multi-parameter flow cytometry

Single peptides identified as containing targeted epitopes were used as the antigenic stimulus. The negative control was media alone and the positive control was staphylococcal enterotoxin B (SEB). A peptide pool consisting of CMV, EBV and Influenza epitopes (CEF) was also used in the assay.

Briefly, cryopreserved PBMC were thawed and cells were stimulated for one hour with either media alone, antigen, or positive control. The antibody, CD107 a/b-PECy5 was added with stimulation. Brefeldin A (Sigma-Aldrich, St. Louis, MI, USA) was then added at a concentration of 5 µg/ml, and cells incubated overnight. The next day, PBMC were washed and stained with antibodies, in different combinations, against CD4-Alexa700, CD8-Pacific Blue, CD45RA-biotin, CCR7-PECy7, CD57-PECy5, CD279(PD-1)-PE, and a live/dead marker emitting in the aqua wavelength, for 20 minutes at 4°C. Cells were washed twice and stained with the secondary antibody streptavidin-Qdot655 for 20 minutes at 4°C. Cells were then washed and fixed in 2% paraformaldehyde. The cells were permeabilized using FACS Perm solution (BD Biosciences), washed and stained using antibodies, in different combinations, against CD3-ECD, IFN-γ-APC, TNF-α-FITC, and MIP-1β-PE for 30 minutes at 25°C. Following staining, the cells were washed, fixed in 1% paraformaldehyde, and collected on a BD LSR-II using FACS DIVA software (BD Biosciences). Data was analyzed using FlowJo software (TreeStar).

### Gating Strategy

In all analyses, forward scatter (FSC)-height versus FSC-area plot was used to exclude all cell conjugates. Dead cells were then excluded by only gating on cells negative for the live/dead marker. A FSC-area vs. side scatter (SSC)-area plot was used to define the lymphocyte gate. T cells were selected by gating on CD3+ lymphocytes, followed by selection of CD8+ cells by gating on CD3+CD8+ cells. CD4+ cells were defined as CD3+CD8− cells. In panels with CD3, CD4, and CD8 antibodies, on average, 93% of CD3+CD8− cells were CD4+. CD57+ cells were defined using a FITC “fluorescence minus one” (FMO) sample. Quadrant gates were set for expression of CCR7 and CD45RA by using a QDot655 FMO and a PECy7 FMO. IFN-γ+ cells were defined using an APC FMO. IFN-γ+ cells were further analyzed for expression of T-cell memory markers in a CCR7 versus CD45RA.

### Statistical Methods

A median (interquartile range) was used as a measure of central tendency for continuous variables. The Mann-Whitney two-tailed t-test was used for all simple comparisons between two groups. The Spearman Rank correlation test and linear regression analyses were used to explore associations between 2 continuous variables. Differences between categorical data were calculated using Fisher's exact test. A p-value of <0.05 was considered significant. All statistical analyses were performed using the GraphPad Prism 4.03 software package (La Jolla, CA).
